# Global burden and risk factors of MASLD: trends from 1990 to 2021 and predictions to 2030

**DOI:** 10.1007/s11739-025-03895-6

**Published:** 2025-02-28

**Authors:** Minshan Huang, Hang Chen, Hui Wang, Yudi Zhang, Liya Li, Yang Lan, Lanqing Ma

**Affiliations:** The First Affiliated Hospital, Yunnan Institute of Digestive Disease, Kunming Medical University, No. 295 Xichang Road, Kunming, 650032 China

**Keywords:** Epidemiology, Mortality, Disability-Adjusted Life Years (DALYs), Public Health Interventions, Bayesian Age-Period-Cohort Model

## Abstract

The prevalence of metabolic dysfunction-associated steatotic liver disease (MASLD) is increasing globally, posing a major public health issue. This study analyzes the global, regional, and national burden of MASLD and its risk factors from 1990 to 2021, with projections to 2030. We obtained data on MASLD prevalence, deaths, and disability-adjusted life years (DALYs) from the Global Burden of Disease 2021 for 204 countries. Counts and rates per 100,000 population were calculated, and trends to 2030 were predicted using the Bayesian Age-Period-Cohort model. In 2021, there were 1,267.9 million MASLD cases globally, with 138.3 thousand deaths and 3667.3 thousand DALYs. The global age-standardized prevalence, death, and DALY rates were 15,018.1, 1.6, and 42.4 per 100,000 population, increasing by 24.3%, 5.5%, and 5.5% since 1990. North Africa and the Middle East had the highest prevalence, while Andean and Central Latin America had the highest death and DALY rates. Men aged 15–69 and 90–94 had higher DALY rates, while women aged 70–89 and 95+ had higher rates. A reversed V-shaped association was found between the sociodemographic index and DALY rate. High fasting plasma glucose (5.9%) and smoking (2.4%) were major risk factors. Public health efforts should prioritize early detection and management of MASLD in younger populations and develop targeted strategies for older adults, especially women, to reduce the disease burden. Effective policies to address high fasting plasma glucose and smoking can mitigate MASLD’s impact.

## Introduction

Non-alcoholic fatty liver disease (NAFLD) affects about 30.05–32.4% of the global population and is a key component of metabolic syndrome [1]. Recently, it has been proposed to be renamed “steatotic liver disease” (SLD) [2], which include various classifications: metabolic dysfunction-associated SLD (MASLD), MASLD with increased alcohol intake, alcohol-related liver disease, SLD with specific etiologies, and cryptogenic SLD. MASLD is characterized by hepatic steatosis along with at least one of five cardiometabolic risk factors of metabolic syndrome [2].

MASLD encompasses a spectrum of liver conditions, from simple steatosis to metabolic-associated steatohepatitis (MASH), which can progress to cirrhosis, liver failure, and hepatocellular carcinoma (HCC) [3, 4]. Strongly associated with metabolic syndrome, obesity, type 2 diabetes, and dyslipidemia, MASLD reflects the impact of modern lifestyle and dietary patterns [5]. The increasing burden of MASLD presents a major public health challenge due to its high prevalence, potential for severe liver-related morbidity and mortality, and the increased risk of cardiovascular mortality.

The Global Burden of Disease (GBD) study from 1990 to 2021 reported the burden of MASLD in 204 countries and territories, categorized by gender, region, and sociodemographic index (SDI) [6]. The GBD database has been crucial for numerous studies evaluating the incidence, prevalence, and mortality rates of liver disease. Analyzing the latest GBD 2021 data helps researchers develop more effective prevention strategies.

This study examines the prevalence, deaths, and disability-adjusted life years (DALYs) associated with MASLD, analyzing disease trends by age and sex across 204 countries and territories from 1990 to 2021 using GBD 2021 data. Additionally, the Bayesian Age-Period-Cohort (BAPC) model was used to predict trends up to 2030 for age-standardized disability-adjusted life years rate (ASDR), age-standardized years of life lost (YLLs), age-standardized years lived with disability (YLDs), and age-standardized mortality rate (ASMR).

By systematically assessing the burden of MASLD at various geographical levels, this study aims to improve understanding of this growing public health issue and guide the development of effective interventions and policies to combat MASLD globally.

## Methods

### Study design

The Global Burden of Disease (GBD) project is one of the most comprehensive and systematic epidemiological studies worldwide. The GBD 2021 study, published in 2024, assessed 369 diseases and injuries and 87 risk factors worldwide [7, 8]. We extracted mortality and population data for MASLD from the GBD 2021 database, stratified by age, sex, year, country, and region. The data is accessible via web-based tools (https://vizhub.healthdata.org/gbd-results/). This study adheres to the Guidelines for Accurate and Transparent Health Estimates Reporting.

### Disease definition

Experts proposed renaming non-alcoholic fatty liver disease (NAFLD) to metabolic dysfunction-associated steatotic liver disease (MASLD) [9]. MASLD is characterized by hepatic steatosis along with at least one of five cardiometabolic risk factors of metabolic syndrome [2]. However, MASLD outcomes were not included in the 2021 GBD database; thus, we used NAFLD data from the 2021 GBD database.

### Risk factors

Strong evidence identifies smoking status and high fasting plasma glucose as key risk factors for MASLD. We also reported the proportion of DALYs attributable to each risk factor.

### Statistical analysis

We present the age-standardized prevalence, death, and DALYs rates of MASLD across 21 regions and 204 countries, along with data by age and sex. The SDI, which ranges from 0 to 1, reflects fertility rates in those under 25, income per capita, and average educational attainment among those over 15 years. Higher SDI values indicate better socioeconomic development. We extracted regional SDIs from the GBD 2021 and used Gaussian process regression to analyze the relationship between SDI and age-standardized DALYs of MASLD by location from 1990 to 2021.

We employed the BAPC model to predict age-standardized disability-adjusted life years rate (ASDR), age-standardized years of life lost (YLLs), age-standardized years lived with disability (YLDs), and age-standardized mortality rate (ASMR). This model has shown superior predictive accuracy compared to models like the Joinpoint and Poisson regression [10]. BAPC analysis was conducted using the integrated nested Laplace approximation. The model assumes an independent normal distribution of the mean of second-order differences of all effects, which smooths age, period, and birth cohort trends, preventing parameter estimates from deviating excessively between adjacent time periods. Detailed model principles and formulas are available in previous studies [10, 11]. All data processing, analysis, and visualization were conducted using R (version 4.3.1).

## Results

### The overall burden of MASLD

#### Global level

In 2021, there were 1267.9 million prevalent cases of MASLD globally, with an age-standardized prevalence rate of 15,018.1 per 100,000, reflecting a 24.3% increase since 1990. MASLD resulted in 138.3 thousand deaths in 2021, with an age-standardized rate of 1.6, a 5.5% increase since 1990. The global number of DALYs due to MASLD in 2021 was 3667.3 thousand, with an age-standardized rate of 42.4 per 100,000, also a 5.5% increase since 1990 (Table 1). Thus, while the age-standardized prevalence rate of MASLD increased significantly from 1990 to 2021, the changes in age-standardized death and DALY rates were less pronounced.

**Table 1:** Age-standardized prevalence, death and DALY rates of MASLD globally and regionally in 1990 and 2021

	Prevalence (95% UI)			Deaths (95% UI)				DALYs (95% UI)		
	No,in thousands (95%UI)	ASRs per 100000 (95%UI)	Percentage change in ASRs from 1990 to 2021	No,in thousands (95%UI)	ASRs per 100000 (95%UI)	Percentage change in ASRs from 1990 to 2021		No,in thousands (95%UI)	ASRs per 100000 (95%UI)	Percentage change in ASRs from 1990 to 2021
Global	1267.9 (1157.9 ;1380.4)	15018.1 (13756.5,16361.4)	24.3 (23.2,25.4)	138.3 (108.3,173.9)	1.6 (1.3,2)	5.5 (−6.9,17.8)		3667.3 (2903.6,4607.3)	42.4 (33.6,53.3)	5.5 (−6.2,16.9)
High-income Asia Pacific	24.7 (22.6 ;26.8)	8885.7 (8148.4,9666.7)	15.5 (13.4,18)	4.5 (3.4,5.7)	0.9 (0.7,1.1)	−44.3 (−49.8,−38)		79.5 (61.4,97.5)	18.7 (14.8,23.1)	−50.6 (−55, −45.4)
High-income North America	49 (44.7 ;53.4)	10056 (9187.3,10925.6)	26.5 (24.2,29.1)	9.8 (7.6,12.4)	1.6 (1.2,2)	45.5 (38.3,55)		241.7 (188.3,307)	42.1 (33,54.2)	39.6 (33.1,48.2)
Western Europe	66.3 (60.9 ;71.6)	10841.8 (9939,11802)	33.1 (31.2,35.3)	15.7 (11.8,19.6)	1.7 (1.3,2.2)	−19.6 (−25.2, −12.8)		349.7 (269.8,441.9)	45.3 (35,57.1)	−22.3 (−27.6, −16.4)
Australasia	3.8 (3.5 ;4.1)	9468.2 (8665.5,10349.4)	28.4 (24.7,32.2)	0.7 (0.5,0.8)	1.3 (1,1.5)	61.9 (40,89)		15.5 (12.4,19)	33.2 (26.6,40.6)	51.5 (32.2,74.4)
Andean Latin America	9.7 (8.9 ;10.6)	14984.8 (13708.4,16361.5)	15.7 (13.2,18.6)	3.4 (2.4,4.7)	5.9 (4,8.1)	24.4 (2,51.7)		86.2 (60.1,120.2)	142.2 (98.6,198.8)	14.2 (−7.2,41.4)
Tropical Latin America	42.9 (39.2 ;46.9)	16662.7 (15244.9,18205.5)	11.1 (8.9,13.6)	3.6 (2.7,4.6)	1.4 (1,1.8)	16.9 (8.9,25)		101.3 (74.6,133.4)	38.6 (28.6,50.5)	11.4 (2.8,19.4)
Central Latin America	44.7 (40.9 ;48.8)	16984 (15536.5,18533.6)	13.5 (11.9,15.2)	12.6 (9.5,16.3)	5 (3.7,6.4)	18.8 (5,33.5)		362.1 (270.5,471.2)	138.8 (104.4,180.6)	20 (6,34.8)
Southern Latin America	8.1 (7.4 ;8.8)	10292.5 (9394.6,11265.4)	28.7 (25,32.4)	1.5 (1.1,2)	1.7 (1.2,2.3)	−5.2 (−12.5,2.4)		36.9 (26.8,50.8)	44 (31.9,60.6)	−11.3 (−18.8, −3.8)
Caribbean	8.1 (7.4 ;8.8)	15650.7 (14340.1,16986.2)	11.2 (9.2,13.3)	1.6 (1.1,2.2)	3 (2.1,4.1)	5.3 (−11.2,23)		42.9 (30,59.4)	80.3 (56.3,110.8)	8.8 (−8.6,27.4)
Central Europe	20.6 (18.8 ;22.4)	12731.5 (11618.6,13852.6)	12 (10.4,13.4)	3.5 (2.6,4.7)	1.7 (1.2,2.3)	23.5 (13.8,32.9)		90.8 (66,123.7)	47.9 (34.8,64.8)	25.8 (16.4,35.6)
Eastern Europe	34.7 (31.7 ;37.8)	12293.9 (11254.5,13359.2)	10.9 (8.5,13.4)	8.4 (6.2,11.4)	2.7 (1.9,3.6)	189.7 (150.8,232.5)		270.2 (193,372.7)	91.4 (65.2,125.3)	247.8 (198.4,298.6)
Central Asia	15.2 (13.9 ;16.7)	16120.1 (14735.3,17604.5)	14.2 (12.3,16.1)	2.8 (2,3.7)	3.4 (2.5,4.6)	56.7 (40.1,73.8)		83.1 (60.2,114.2)	92.2 (67.2,125)	59.2 (40.7,79.1)
North Africa and Middle East	164.3 (151.4 ;179.1)	27686.7 (25586.9,29914.6)	26.4 (24.5,28.4)	11 (8,15)	2.7 (1.9,3.7)	2 (−19.5,28.6)		276.3 (205.3,374.1)	59 (43.6,79.8)	9.3 (−12.8,35.4)
South Asia	249.8 (227.9 ;273.2)	14158.3 (12940.9,15445.1)	14.5 (13.3,15.8)	18.7 (13.6,24.9)	1.3 (0.9,1.7)	17.9 (−20.7,59.4)		540.6 (397.4,722.3)	33.7 (24.7,44.6)	10.7 (−22.4,43.9)
Southeast Asia	115.1 (104.8 ;125.9)	15691.7 (14308.3,17127.2)	12.2 (10.9,13.6)	11.5 (8.4,15)	1.9 (1.3,2.4)	14.8 (−17.3,51.8)		313.3 (235.1,411.9)	45.3 (34,58.8)	9 (−19.1,40.2)
East Asia	301.4 (274.4 ;328.8)	15596.2 (14262.4,16999.3)	21.9 (19.9,24.3)	17.6 (13.6,22)	0.8 (0.7,1)	−20.1 (−37.2,2.5)		435.2 (336.7,552.1)	20.1 (15.6,25.1)	−26.7 (−42.2, −5.3)
Oceania	1.6 (1.5 ;1.8)	15182.7 (13936.2,16584.7)	10.8 (8,13.1)	0.1 (0,0.1)	0.8 (0.6,1.1)	−9.9 (−29.9,16.3)		2.1 (1.5,2.9)	22.1 (15.7,31.1)	−11.8 (−31.2,15.3)
Western Sub-Saharan Africa	48 (43.9 ;53)	14936.8 (13659.3,16347.6)	12.2 (11,13.5)	5.1 (3.8,6.7)	2.8 (2.1,3.6)	0 (−28.4,31.6)		148.9 (110.2,198.1)	65.3 (49.3,86.3)	−1.9 (−28.4,28.7)
Eastern Sub-Saharan Africa	37.3 (34 ;41.5)	13162.1 (12037.1,14400.2)	11.8 (10.5,13.2)	3.8 (2.9,5)	2.4 (1.8,3.2)	5.1 (−10.4,22.2)		113.5 (85.9,151.4)	57.5 (43,76.3)	2.7 (−11,19.4)
Central Sub-Saharan Africa	10.9 (9.8 ;12)	11870.6 (10844.9,12943.4)	8.9 (5.8,12.2)	0.9 (0.6,1.4)	1.6 (1,2.5)	−8.5 (−28.6,15.2)		30.5 (20,45.9)	43.1 (28.2,65.8)	−9.7 (−29.8,13.8)
Southern Sub-Saharan Africa	11.8 (10.8 ;12.9)	15937.2 (14572.6,17388)	14.4 (12.5,16.7)	1.6 (1.2,2)	2.8 (2.2,3.5)	52.5 (7.6,108.9)		47.1 (37.3,59.7)	72.7 (57.6,91.1)	46.6 (6.1,95.2)

*DALY* Disability-adjusted life year

#### Regional level

In 2021, the highest age-standardized prevalence of MASLD per 100,000 was observed in North Africa and the Middle East (27,686.7), while the lowest was in High-income Asia Pacific (8885.7), Australasia (9468.2), and High-income North America (10,056) (Table 1). The highest age-standardized death rates were in Andean Latin America (5.9) and Central Latin America (5), with the lowest in Oceania (0.8), East Asia (0.8), and High-income Asia Pacific (0.9) (Table 1). Andean Latin America (142.2) and Central Latin America (138.8) had the highest age-standardized DALY rates per 100,000, whereas High-income Asia Pacific (18.7), East Asia (20.1), and Oceania (22.1) had the lowest (Table 1).

The most significant increases in the age-standardized prevalence of MASLD from 1990 to 2021 were observed in Western Europe (33.1%), Southern Latin America (28.7%), and Australasia (28.4%), with the smallest increases in Central Sub-Saharan Africa (8.9%), Oceania (10.8%), and Eastern Europe (10.9%) (Table 1). During this period, the largest increases in age-standardized death rates were in Eastern Europe (189.7%), Australasia (61.9%), and Central Asia (56.7%), while the largest decreases were in High-income Asia Pacific (−44.3%) and Western Europe (−19.6%) (Table 1). The greatest increases in age-standardized DALY rates were in Eastern Europe (247.8%), Central Asia (59.2%), and Australasia (51.5%), with the largest decreases in High-income Asia Pacific (−50.6%), East Asia (−26.7%), and Western Europe (−22.3%) (Table 1).

#### National level

**Fig. 1:**
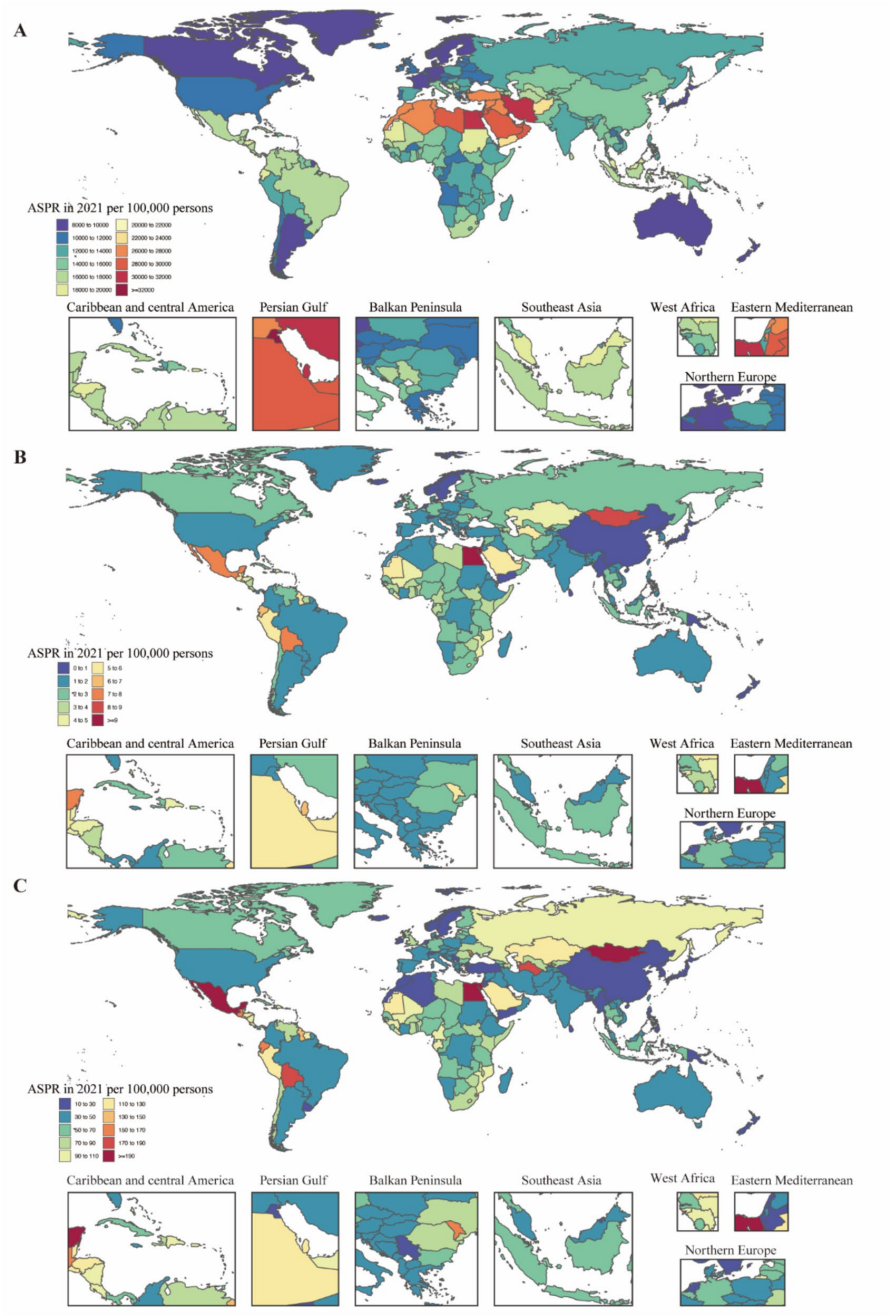
Age-standardized prevalence, death and DALY rates attributable to MASLD for both sexes combined in 2021. **A** Prevalence. **B** Deaths. **C** DALYs. *DALY* Disability-adjusted life year

In 2021, the national age-standardized point prevalence of MASLD ranged from 8133.47 to 32,312.17 cases per 100,000. Kuwait (32,312.17), Egypt (31,668.80), and Qatar (31,327.55) had the highest age-standardized prevalences, while Japan (8133.47), Finland (8358.51), and Canada (8492.32) had the lowest (Fig. 1A). The national age-standardized death rates for MASLD in 2021 varied from 0.43 to 9.44 deaths per 100,000, with the highest rates in Egypt (9.44), Mongolia (8.64), and Mexico (7.24), and the lowest in Papua New Guinea (0.43), Singapore (0.51), and Sri Lanka (0.56) (Fig. 1B). In 2021, the national age-standardized DALY rate of MASLD ranged from 10.64 to 201.86 per 100,000. The highest rates were in Mexico (201.86), Egypt (196.55), and Mongolia (195.28), while the lowest rates were in Singapore (10.64), Papua New Guinea (12.28), and Sri Lanka (13.76) (Fig. 1C).

### Age and sex patterns

**Fig. 2:**
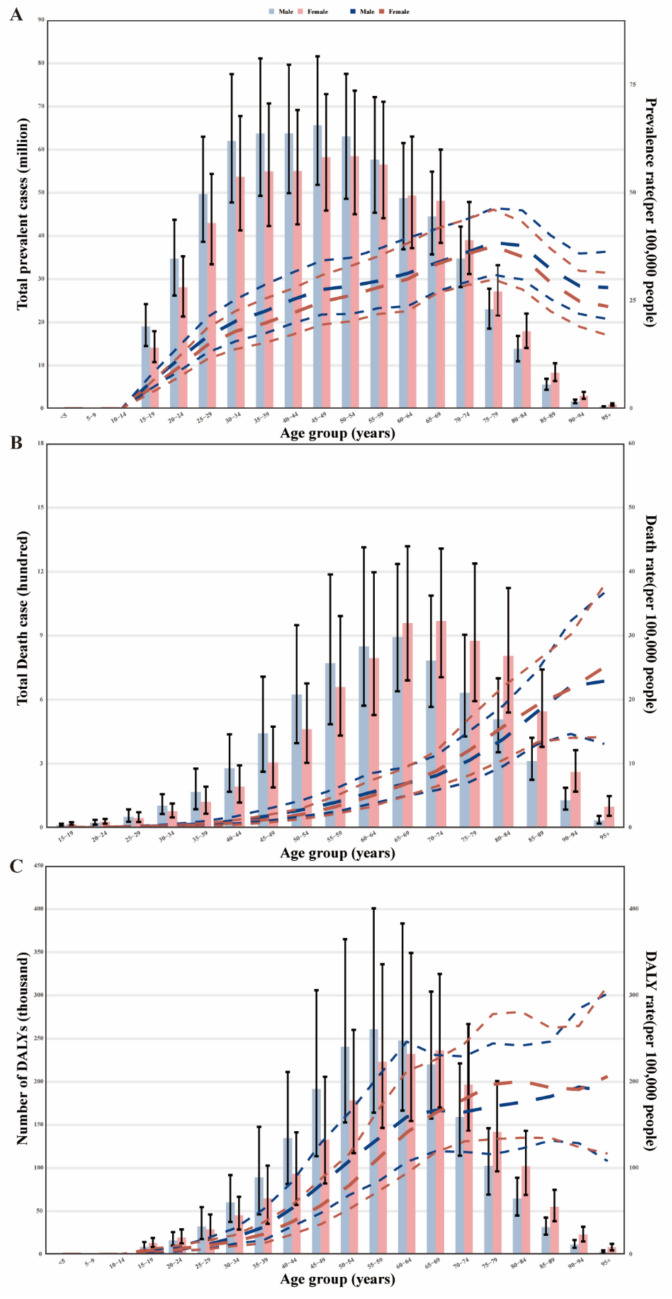
Age-specific numbers and rates of prevalence, deaths and DALYs attributable to MASLD by sex, in 2021. **A** Prevalence. **B** Deaths. **C** DALYs. *DALY* Disability-adjusted life year

In 2021, the global prevalence rate of MASLD began increasing in the 10–14 age group and peaked in the 75–79 age group. The number of prevalent cases was highest in the 45–49 age group before declining with age. Prevalent cases were higher in men up to the 55–59 age group, while MASLD was more common in women over 60 years (Fig. 2A). The MASLD death rate increased with age, with higher rates in males across the 15–64 age groups. The rates intersect in the 65–69 and 90–94 age groups, with higher rates in females in the 70–90 and 95+ age groups. Deaths peaked in the 65–69 age group for males and the 70–74 age group for females, then decreased with age. Deaths from MASLD were higher in men aged 25–64 years, while women had higher rates in the 15–24 and 65+ age groups (Fig. 2B). The global DALY rate for MASLD increased with age, peaking in the 55–59 age group. The rate was higher in men across the 15–69 and 90–94 age groups but higher in women in the 70–89 and 95+ age groups. The number of DALYs peaked in the 55–59 age group (Fig. 2C). DALYs were higher in men aged 25–64 years but more common in women younger than 24 and older than 65 years.

### Association with the sociodemographic index

**Fig. 3:**
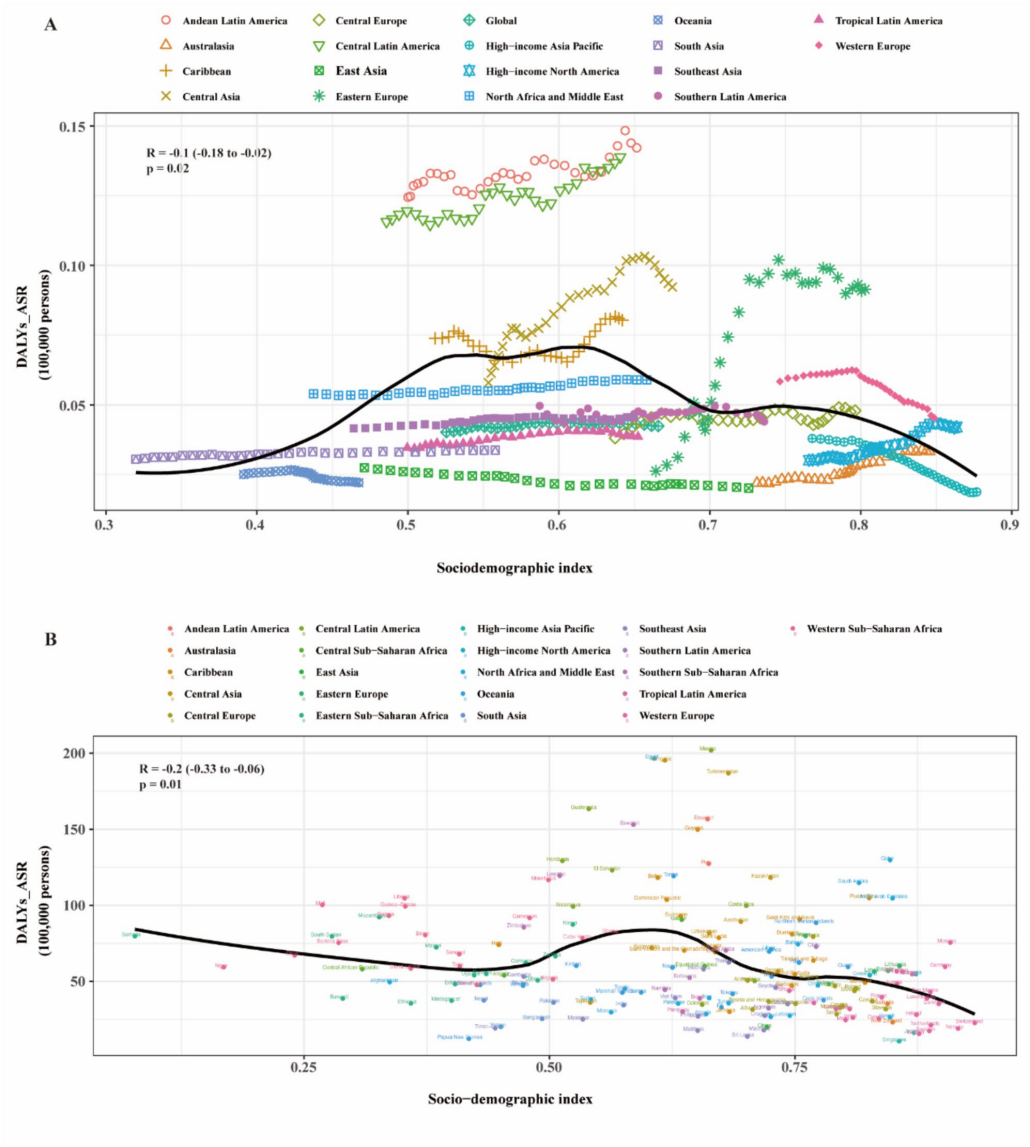
Age-standardized DALY rates attributable to MASLD across 21 GBD regions by Sociodemographic Index for both sexes combined, 1990–2021. **A** For each region, points from left to right depict estimates from each year from 1990 to 2021. **B** Age-standardized DALY rates attributable to MASLD across 195 countries and territories by Sociodemographic Index for both sexes combined in 2021. *DALY* Disability-adjusted life year; *GBD* Global Burden of Disease Study

At the regional level, a reversed V-shaped association was found between the sociodemographic index (SDI) and the age-standardized DALY rate of MASLD from 1990 to 2021 (Fig. 3A). Expected values, based on SDI and disease rates in all locations, are shown as a solid line. The age-standardized DALY rate increased with SDI up to about 0.62, then decreased. Generally, the age-standardized DALY rate of MASLD and SDI were negatively correlated. Andean Latin America, Central Latin America, and Western Europe had higher than expected DALY rates based on their SDI, whereas Oceania, East Asia, Southeast Asia, Tropical Latin America, and High-income Asia Pacific had lower than expected burdens from 1990 to 2021 (Fig. 3A).

In 2021, the national burden of MASLD decreased with increasing socioeconomic development before an SDI of about 0.43 and after an SDI of about 0.62, but increased between an SDI of about 0.43 and 0.62. Countries such as Egypt, Mexico, and Turkmenistan had much higher than expected burdens, while Singapore and Sweden had much lower than expected burdens (Fig. 3B).

### Risk factors

**Fig. 4:**
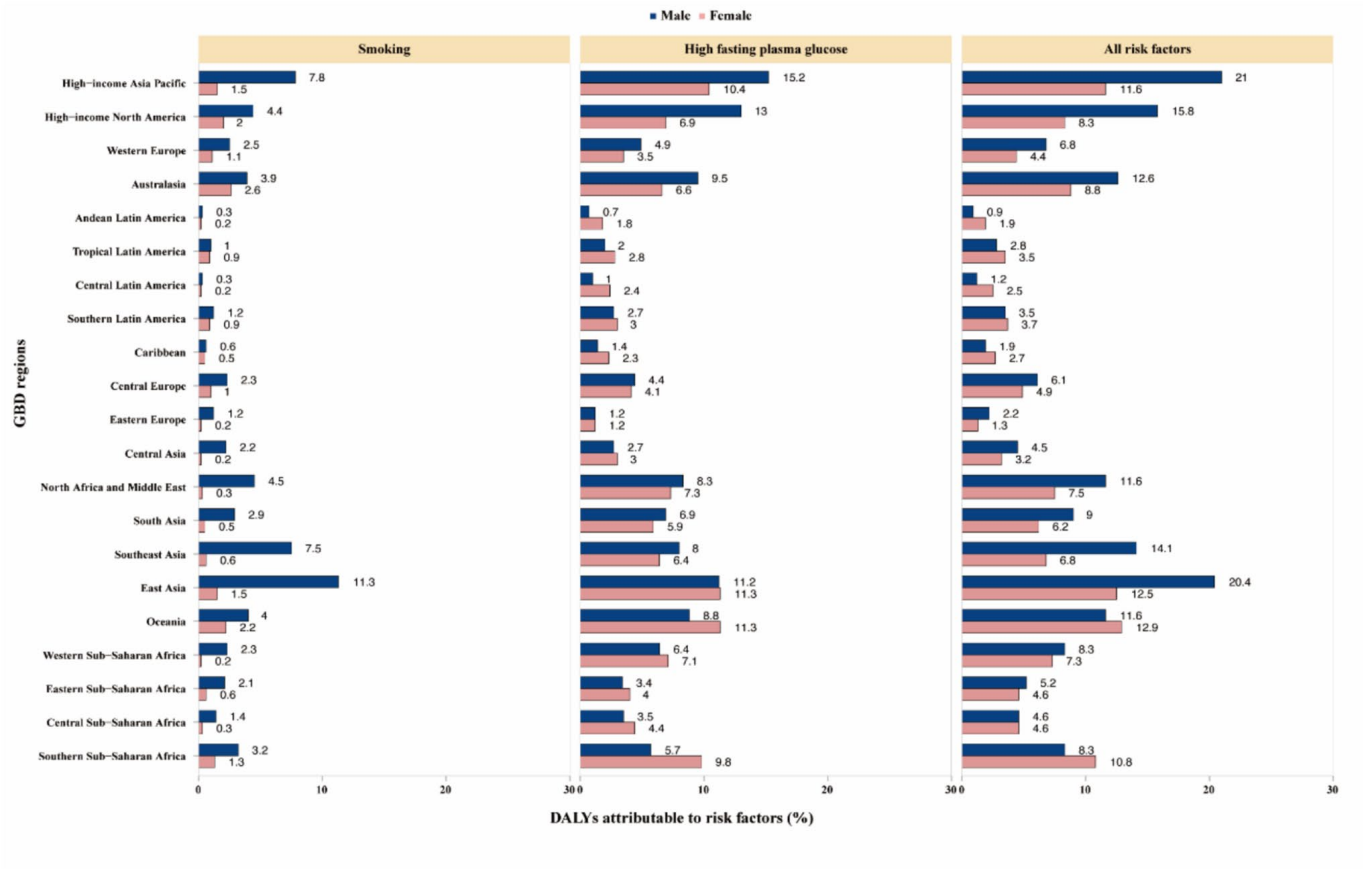
High fasting plasma glucose and smoking age-standardized DALYs are major risk factors for MASLD by region for females and males in 2021. *DALY* Disability-adjusted life year, *GBD* Global Burden of Disease Study

The proportion of DALYs due to MASLD attributable to individual risk factors varied across Global Burden of Disease regions. High fasting plasma glucose (5.9%) and smoking (2.4%) significantly contributed to DALYs due to MASLD (Fig. 4). The proportion of DALYs attributable to these risk factors was generally higher in men, with high fasting plasma glucose contributing more significantly than smoking.

### Bayesian age-period-cohort analysis prediction model

**Fig. 5:**
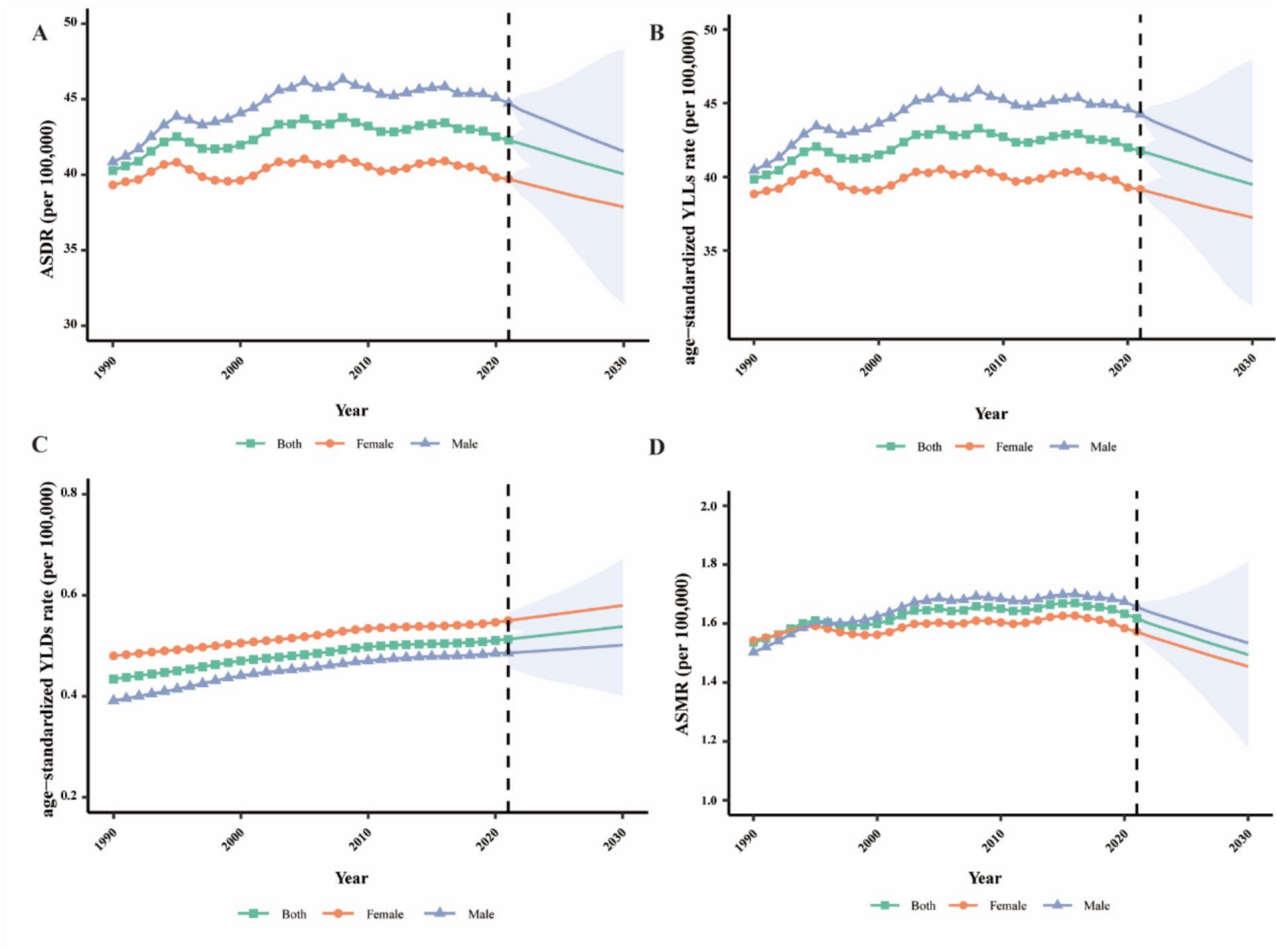
The global burden of MASLD. **A** ASDR by sex from 1990 to 2030. **B** YLLs by sex from 1990 to 2030. **C** YLDs by sex from 1990 to 2030. **D** ASMR by sex from 1990 to 2030. *ASDR* Age-standardized disability-adjusted life years rate, *YLLs* Years of life lost, *YLDs* Years lived with disability, *ASMR* Age-standardized mortality rate

Globally, the MASLD-related age-standardized disability-adjusted life years rate (ASDR) showed an upward trend from 1990 (40.28 per 100,000 [95% CI 40.20 to 40.36]) to 2021 (42.28 per 100,000 [95% CI 42.34 to 42.22]) (Fig. 5A). There was also an increase in the YLLs rate (4.84%) and YLDs rate (18.2%) (Fig. 5B and C). ASDR and age-standardized YLLs rates are projected to decline slowly over the next decade, while age-standardized YLDs rates will rise slightly. Additionally, the age-standardized mortality rate (ASMR) (5.2%) showed an upward trend. Projections suggest that ASDR and ASMR will slowly decrease in the next ten years (Fig. 5D). However, the modest upward trend in age-standardized YLDs rates is not expected to abate in the next decade. Men generally exhibited higher ASDR and ASMR with stable trends compared to women, indicating that the MASLD-related burden has been and will continue to be greater among men, despite future declines in ASDR and ASMR.

## Discussion

Our results found that the age-standardized prevalence rate of MASLD increased by 24.3% from 1990 to 2021, while age-standardized rates for deaths and DALYs both rose by 5.5%. This increase trend consistent with 2019 findings, reflects a growing global health burden that demands urgent attention and effective intervention strategies.

Kuwait, Egypt, and Qatar have the highest prevalence rates, which means these areas could face serious public health challenges, and need more resources to management and treatment of patients with MASLD. Except for the influence of factors such as urbanization, increased sedentary behavior, global shift to westernized diets high in sugar, fat, and processed foods, high prevalence may primarily due to the lack of effective public health policies in these regions has led to high rates of obesity and type 2 diabetes because of poor management, thus increasing the prevalence of MASLD [1, 12, 13]. Conversely, Japan, Finland, and Canada exhibit the lowest prevalence rates of MASLD. It indicates that these countries may have advantages in public health management and health policy, and these factors may have reference significance for other regions. Japan and Finland have robust policies and programs like Specific Health Check-ups [14] and Wellbeing from Healthy Nutrition and Physical Activity [15], for managing obesity and diabetes, resulting in lower increases in MASLD prevalence. Additionally, these high SDI countries typically possess advanced healthcare systems that facilitate extensive health screening and disease management, ensuring early diagnosis and timely treatment [16, 17]. Their comprehensive healthcare systems and high public health awareness contribute significantly to low MASLD prevalence rates.

In addition, we found poor concordance between the prevalence and death rates or DALY rates attributable to MASLD. For example, Bolivia and Peru have relatively low MASLD prevalence, ranking 137th and 125th out of 204 countries, respectively. However, their DALY and mortality rates are disproportionately high, with Bolivia ranking 5th in DALY rates and 4th in mortality rates, and Peru ranking 14th in both. This indicates that there are significant challenges in addressing MASLD in these countries, including inadequate screening, diagnosis, and treatment of MASLD, which results in many cases remaining undiagnosed until the disease progresses to more severe stages. This phenomenon can result from a combination of inadequate health care resources, weak public health infrastructure, socioeconomic inequities, cultural and lifestyle factors, and limitations in data collection and reporting systems. This finding was more evident in low-income countries where healthcare services are designed to treat acute conditions, such as infectious disorders, and are not appropriately developed to manage chronic diseases. Conversely, Kuwait and Sri Lanka have relatively high MASLD prevalence, ranking 1st and 66th out of 204 countries, respectively, but their DALY and mortality rates are disproportionately low. Specifically, Kuwait ranks 181st in DALY rates and 160th in mortality rates, while Sri Lanka ranks 202nd in both. This discrepancy is likely because of improvements in diagnostic measures and better management of MASLD and related comorbidities.

Our data revealed a reversed V-shaped association between the sociodemographic index (SDI) and the age-standardized DALY rate of MASLD from 1990 to 2021. The SDI serves as a comprehensive indicator of poverty. Countries with low to mid-range SDI values, such as Bolivia, Peru, Nigeria, and Ethiopia, often prioritize infrastructure and economic growth over healthcare system development. Public health policies and programs for preventing and managing chronic diseases are lacking, and lower education levels lead to limited health awareness. Additionally, these regions face the dual challenges of rising obesity rates and insufficient healthcare infrastructure, hindering effective disease management. Economic disparities also limit access to healthcare services and interventions, exacerbating the burden. In summary, countries with lower sociodemographic index scores typically experience limited access to healthcare services. These systemic inadequacies significantly contribute to the high disease burden observed in patients with MASLD.

Although the prevalence of MASLD increased with age, the affected population showed a younger trend, and the prevalence was higher in men, While the deaths and DALYs were mainly in middle-aged and elderly people. These findings indicate the disease burden of MASLD in different age and gender groups and provide new perspectives for global health research. Study results highlight the importance of considering age and sex differences when developing public health strategies. The high mortality and DALY rates in young and middle-aged men suggest the need for specific interventions, while the high prevalence and mortality rates in older women indicate the need for health management of the aging population. In a word, public health initiatives should focus on the early detection and management of MASLD in younger populations, as well as targeted strategies for older persons, and should also consider gender factors to reduce the overall burden of disease.

High fasting plasma glucose and smoking are significant contributors to DALYs associated with MASLD, with a more pronounced impact in men. The higher proportion of DALYs due to high fasting plasma glucose reflects the strong link between hyperglycemia, insulin resistance, and liver fat accumulation [18–20], while the contribution of smoking highlights the role of oxidative stress and inflammation in liver disease progression [21]. Addressing these risk factors through targeted public health interventions, such as promoting healthy diets to manage blood glucose levels and anti-smoking campaigns, is crucial for reducing the global burden of MASLD.

In summary, this study highlights the substantial and growing burden of MASLD across different regions and demographics. Public health initiatives should focus on early detection and management of MASLD in younger populations, and targeted strategies for older adults, particularly women, to reduce the disease burden. Addressing high fasting plasma glucose and smoking through effective policies can mitigate MASLD's impact. Integrating the latest technological advances and research findings into public health strategies will be essential for effectively combating this global health challenge.

## Strengths and limitations of this study

A strength of this study is that it provides comprehensive estimates of MASLD levels and trends, as well as its risk factors, at global, regional, and national levels from 1990 to 2021. Additionally, the BAPC model was used to predict trends of ASDR and ASMR for the next nine years. However, this study has several limitations: (1) The quality of the data used in this study is contingent upon the quality control measures implemented during the original data collection process, which inevitably affects the accuracy of studies in some regions. Despite the use of robust statistical methods, under-registration of liver disease cases and insufficient local health resources can lead to misdiagnosis or underdiagnosis. (2) Some risk factors, such as genetic predisposition, although rare, could not be taken into account in our estimations. This limitation means that the study may not fully capture all the risk factors contributing to MASLD. (3) Many countries lack efficient systems for registering deaths, so verbal autopsy studies were relied on for death estimates. Improving vital registration systems might enhance the accuracy of these data and allow better management of the disease burden. Further research is needed to uncover the full disease burden associated with MASLD. (4) It is important to note that our study utilized NAFLD data, as MASLD has only recently been introduced in the literature. Therefore, the results may be more applicable to NAFLD, and caution should be taken when generalizing these findings to MASLD. (5) The study primarily focuses on the prevalence of MAFLD across different age groups and does not analyze trends in disease incidence or prevalence since 1990. Future studies should incorporate long-term data on both incidence and diagnostic trends to better understand the underlying factors contributing to the rise in prevalence.

## Data Availability

Data will be made available on request.

## Abbreviations

MASLD: Metabolic Dysfunction-Associated Steatotic Liver Disease
NAFLD: Non-Alcoholic Fatty Liver Disease
ASDR: Age-Standardized Disability-Adjusted Life Years Rate
ASMR: Age-Standardized Mortality Rate
BAPC: Bayesian Age-Period-Cohort
DALYs: Disability-Adjusted Life Years
GBD: Global Burden of Disease
SDI: Sociodemographic Index
T2DM: Type 2 Diabetes Mellitus
YLDs: Years Lived With Disability
YLLs: Years of Life Lost
